# Deciphering the Prognostic Efficacy of MRI Radiomics in Nasopharyngeal Carcinoma: A Comprehensive Meta-Analysis

**DOI:** 10.3390/diagnostics14090924

**Published:** 2024-04-29

**Authors:** Chih-Keng Wang, Ting-Wei Wang, Chia-Fung Lu, Yu-Te Wu, Man-Wei Hua

**Affiliations:** 1 School of Medicine, College of Medicine, National Yang-Ming Chiao Tung University, Taipei 112304, Taiwan; 2 Department of Otolaryngology-Head and Neck Surgery, Taichung Veterans General Hospital, Taichung 407219, Taiwan; 3 Institute of Biophotonics, National Yang-Ming Chiao Tung University, 155, Sec. 2, Li-Nong St. Beitou Dist., Taipei 112304, Taiwan; 4 Department of Biomedical Imaging and Radiological Sciences, National Yang-Ming Chiao Tung University, Taipei 112304, Taiwan; alvin4016@ym.edu.tw

**Keywords:** radiomics, prognostic models, meta-analysis, survival

## Abstract

This meta-analysis investigates the prognostic value of MRI-based radiomics in nasopharyngeal carcinoma treatment outcomes, specifically focusing on overall survival (OS) variability. The study protocol was registered with INPLASY (INPLASY202420101). Initially, a systematic review identified 15 relevant studies involving 6243 patients through a comprehensive search across PubMed, Embase, and Web of Science, adhering to PRISMA guidelines. The methodological quality was assessed using the Quality in Prognosis Studies (QUIPS) tool and the Radiomics Quality Score (RQS), highlighting a low risk of bias in most domains. Our analysis revealed a significant average concordance index (c-index) of 72% across studies, indicating the potential of radiomics in clinical prognostication. However, moderate heterogeneity was observed, particularly in OS predictions. Subgroup analyses and meta-regression identified validation methods and radiomics software as significant heterogeneity moderators. Notably, the number of features in the prognosis model correlated positively with its performance. These findings suggest radiomics’ promising role in enhancing cancer treatment strategies, though the observed heterogeneity and potential biases call for cautious interpretation and standardization in future research.

## 1. Introduction

Nasopharyngeal carcinoma (NPC) exhibits notable epidemiological differences globally, with a significantly higher incidence in East and Southeast Asia compared to Western countries. These disparities are attributed to genetic susceptibility, environmental factors, and Epstein-Barr virus (EBV) infection prevalence. The distinct epidemiological patterns of NPC necessitate tailored approaches in diagnosis, treatment, and prognosis across different populations [1,2]. In pursuing personalized medicine, radiomics and machine learning have emerged as transformative tools, offering new avenues for the prognostic assessment of NPC [3,4].

Radiomics involves extracting high-dimensional data from medical images, which, when analyzed through machine learning algorithms, can reveal patterns indicative of tumor phenotype, aggressiveness, and likely response to treatment. This methodology extends the value of conventional MRI scans beyond anatomical visualization, enabling the quantification of tumor heterogeneity at a microscopic level that may not be visually apparent [5,6]. Machine learning further enhances this process by identifying complex relationships between radiomic features and clinical outcomes, facilitating the development of predictive models for NPC prognosis [7,8].

The integration of radiomics and machine learning in NPC research holds the potential to revolutionize patient care. By accurately predicting treatment outcomes, these technologies can guide the selection of therapeutic strategies tailored to individual patient profiles, thus improving survival rates and quality of life. Moreover, the ability to monitor tumor response non-invasively through advanced imaging analytics could lead to more dynamic and responsive treatment plans, adjusting to changes in tumor behavior over time [9,10,11,12].

Despite the promising prospects of radiomics and machine learning in enhancing the prognosis of nasopharyngeal carcinoma (NPC), significant challenges in the standardization of image acquisition, feature extraction, and model validation persist. These hurdles must be overcome to fully leverage the clinical potential of these advanced technologies. A recent meta-analysis highlighted the efficacy of MRI radiomics in predicting the progression-free survival in NPC, presenting a pooled concordance index (C-index) of 0.762 (95% CI, 0.687–0.837) [13]. However, this analysis also noted a high level of heterogeneity (I^2^ = 89%) due to the amalgamation of various endpoints, such as Local Recurrence-Free Survival, Distant Metastasis-Free Survival, and Progression-Free Survival. Our research aims to provide an updated synthesis of the current evidence while offering separate analyses for different endpoints. This approach intends to deliver a more nuanced and comprehensive analysis, potentially reducing heterogeneity and enhancing the interpretability of radiomics in the prognosis of NPC.

## 2. Materials and Methods

This investigation was executed adhering to the Preferred Reporting Items for Systematic Reviews and Meta-Analyses (PRISMA) guidelines for meta-analysis [14]. The PRISMA checklists can be found in Supplemental Table S1. Registration of the study was completed in INPLASY with the registration number INPLASY202420101. It was determined that approval from an ethical review board or obtaining participant informed consent was not requisite for this study.

### 2.1. Database Searches and the Identification of Eligible Manuscripts

Two independent researchers (C-KW and T-WW) conducted an exhaustive literature review, employing a detailed search strategy across PubMed, Embase, and Web of Science, as outlined in Supplementary Table S2. This review spanned the inception of these databases to 17 February 2024. Articles were systematically screened for relevance based on titles and abstracts, with the inclusion and exclusion criteria established collaboratively. Reference lists of key review articles, including [13], were examined to ensure completeness and supplemented by manual searches to capture any overlooked studies. Discrepancies regarding study inclusion were resolved through consultation with a third investigator.

### 2.2. Inclusion and Exclusion Criteria

The inclusion criteria were specified for participants definitively diagnosed with nasopharyngeal carcinoma (NPC), focusing exclusively on adult populations of both sexes. The required imaging criteria stipulated that subjects must have undergone magnetic resonance imaging (MRI) for initial radiomic assessment. This criterion applied to both individuals receiving a new diagnosis and those previously subjected to medical interventions such as surgery, radiation, or chemotherapy. Only studies that included the concordance index (c-index) were considered. The c-index, a measure of the prognostic accuracy of models in time-to-event analysis where data may be censored, was selected based on its utilization in prior research [15], owing to its advantage in providing consistent results across studies with variable endpoints, in contrast to the time-independent area under the curve (AUC) which may lead to heterogeneous outcomes. All observational studies, including retrospective or prospective studies, were included. 

The exclusion criteria were established: studies concerning cancers other than NPC; research employing deep learning-based radiomics, attributed to its lower interpretability; research incorporating multiple timepoint radiomics; individual radiomics feature prediction of prognosis without intergradation with a model; radiomics models incorporating clinical features, not radiomics studies; overlapping datasets; and documents in letters, conference proceedings, retracted papers, or those devoid of images. Further, studies using imaging modalities other than MRI, covering topics or outcomes irrelevant to the study aims, presenting data unsuitable for quantitative analysis, or not reporting the c-index were excluded.

### 2.3. Methodological Quality Appraisal 

The methodological integrity of each study incorporated in the analysis was meticulously assessed via two established instruments: the Quality in Prognosis Studies (QUIPS) tool and the Radiomics Quality Score (RQS) [16,17]. The Quality in Prognosis Studies (QUIPS) tool is employed to rigorously assess the risk of bias across various domains in prognostic studies, each evaluated with specific criteria to ascertain the risk level. Study Participation examines how representative the sample is of the target population, focusing on recruitment efficacy and the demographic and clinical similarity to the broader population. Study Attrition assesses the completeness of the follow-up, scrutinizing follow-up rates and the reasons for dropout to determine potential biases if outcomes for those lost differ from those who completed the study. Prognostic Factor Measurement evaluates the accuracy and consistency with which prognostic factors are measured across participants, emphasizing the method’s reliability and uniform application. Outcome Measurement investigates the reliability and validity of outcome assessments, ensuring clarity in definitions and uniformity in measurement methods. Study Confounding involves identifying and adjusting for potential confounders, assessing the adequacy of their measurement and control. Statistical Analysis and Reporting reviews the appropriateness of statistical methods and the integrity of result reporting. Each domain’s risk of bias is rated as low, moderate, or high, guiding the overall evaluation of a study’s methodological soundness and result reliability.

The RQS, tailored for scrutinizing radiomics research, comprises 16 elements to assess the research’s reliability and susceptibility to bias. Each element within a study was evaluated and scored, leading to an aggregate score representing the sum of scores across all components, reflecting the overall methodological quality of the radiomics studies under review.

### 2.4. Definitions of Prognostic Endpoints

Local Recurrence-Free Survival (LRFS): Constitutes a composite metric evaluating the efficacy of therapeutic interventions in maintaining control at both the primary tumor site and regional levels.

Distant Metastasis-Free Survival (DMFS): Denotes the interval from the commencement of therapeutic measures to the first instance of distant metastasis or death, whichever occurs first, indicating the treatment’s capacity to inhibit tumor dissemination to distal anatomical sites.

Progression-Free Survival (PFS), Disease-Free Survival (DFS), and Failure-Free Survival (FFS): Although these terms are sometimes utilized interchangeably within the oncological lexicon, they predominantly refer to the duration from treatment initiation to the onset of tumor progression, recurrence, or mortality. These measures are critical for assessing the period during which a patient remains unaffected by worsening or reemergence of the disease. For the objectives of this research, these indicators are collectively considered under the umbrella of PFS.

Overall Survival (OS): This parameter measures the time elapsed from the beginning of treatment to death attributable to any cause, serving as a fundamental criterion for evaluating the overall effectiveness of cancer treatment modalities.

### 2.5. Data Extraction and Management 

Data extraction was carried out independently by two authors (C-KW and T-WW), encompassing demographic details, study methodology, and specifics of MRI imaging, as well as radiomics and prognostic model characteristics. This process of data collection, conversion, and amalgamation of the results was executed in alignment with the guidelines stipulated in the Cochrane Handbook for Systematic Reviews of Prognosis Studies and pertinent previous studies.

### 2.6. Statistical Analysis

To address the variability inherent in the studies selected, we employed a random-effects meta-analytical model [18], with statistical significance predetermined at a *p*-value of less than 0.05 (two-tailed). Our findings were visually synthesized in forest plots. During the preliminary exploration phase, we incorporated all outcomes associated with radiomics across various endpoints. The analysis was stratified according to specific endpoints (LRFS, DMFS, PFS, and OS). Concordance indices derived from composite models (those incorporating radiomic features in conjunction with clinical or other model variables) were excluded from the analysis due to the inclusion of varying clinical features across studies, which may introduce substantial heterogeneity [13]. Sensitivity analysis was carried out with the leave-one-out method. In a more focused secondary analysis, attention was narrowed to the Overall Survival endpoint, recognizing its heterogeneity across the studies. Subgroup analyses were conducted based on geographical location (Asian versus Europe), validation methodology (internal validation versus external validation), MRI sequence (single versus multiple), and radiomics software (in-house versus Pyradiomics), alongside meta-regressions on publication year, training sample size, and the number of features used. The consistency of results across the studies was assessed using the Q-test, with a *p*-value less than 0.05 indicating significant heterogeneity. The extent of this heterogeneity was evaluated using I^2^ metrics, categorized as negligible (0–25%), low (26–50%), moderate (51–75%), or high (76–100%) [19]. Vigilance for potential publication bias was maintained, employing the Egger’s method as a diagnostic tool for identifying asymmetry in funnel plots [20]. All statistical analyses were conducted utilizing STATA software (Stata/SE 18.0 for Mac).

## 3. Results

### 3.1. Study Identification and Selection

**Figure 1:**
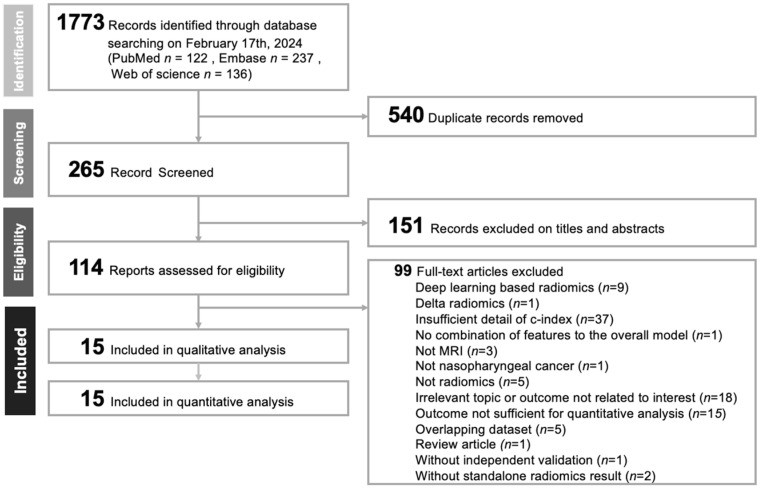
PRISMA flowchart for the current meta-analysis.

Figure 1 displays the PRISMA flow diagram, outlining our systematic review and selection methodology. Initially, 495 studies were identified across multiple databases: 122 from PubMed, 237 from EMBASE, and 136 from Web of Science. The removal of 230 duplicates left 265 articles for initial review. Using Endnote, titles and abstracts were assessed, excluding 151 articles. The remaining 114 articles underwent thorough full-text evaluation. The allocation of articles from each database and the refinement process to identify relevant studies are detailed in Table S2. After comprehensive analysis, 15 studies were included in our meta-analysis [21,22,23,24,25,26,27,28,29,30,31,32,33,34,35], with the exclusion rationale documented in Table S3 [8,10,34,36,37,38,39,40,41,42,43,44,45,46,47,48,49,50,51,52,53,54,55,56,57,58,59,60,61,62,63,64,65,66,67,68,69,70,71,72,73,74,75,76,77,78,79,80,81,82,83,84,85,86,87,88,89,90,91,92,93,94,95,96,97,98,99,100,101,102,103,104,105,106,107,108,109,110,111,112,113,114,115,116,117,118,119,120,121,122,123,124,125,126,127,128,129,130].

### 3.2. Basic Characteristics of Included Studies

A total of 15 studies involving 6243 patients were included. Among these, the majority were conducted in China, with one study from Thailand [21] and another from Italy [32]. The endpoint measures included Local Recurrence-Free Survival (LRFS), Distant Metastasis-Free Survival (DMFS), Progression-Free Survival (PFS), Disease-Free Survival (DFS), Time to Treatment Failure (TTF), and Overall Survival (OS). For details on validation methods, study design, duration, patient demographics, staging, and treatment, refer to Table 1. Information on MRI protocols, such as slice thickness, magnetic field strength, sequences, and scanner types, is available in Table 2. Details on tumor segmentation software, annotators, radiomics software, features, prognostic models, and performance are provided in Table 3.

### 3.3. Methodological Quality of the Included Studies

**Figure 2:**
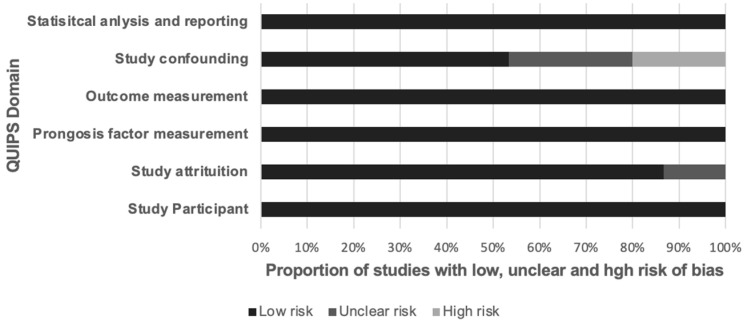
The results of QUIPS quality assessment for included studies.

In assessing the methodological quality of the included studies, we found that the majority exhibited a low risk of bias in the sample size domain as per the Quality in Prognosis Studies (QUIPS) tool. However, approximately 13.3% (2 out of 15) demonstrated some risk of bias in the study attrition domain, and nearly 46.7% (7 out of 15) showed some risk in the confounding domain (see Figure 2). Studies identified as having some risk of bias exhibited protocol variations, potentially influencing the adherence to and outcomes of the prognostic models. Detailed assessments of bias risk using QUIPS and the Radiomics Quality Score (RQS) are documented in Tables S4 and S5.

### 3.4. Primary Outcome: Overall Radiomics Prognosis Model Performance

**Figure 3:**
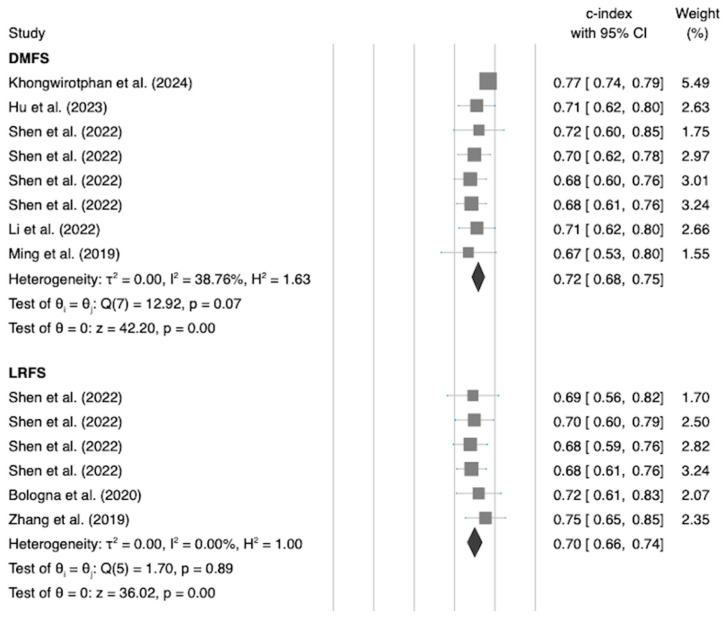

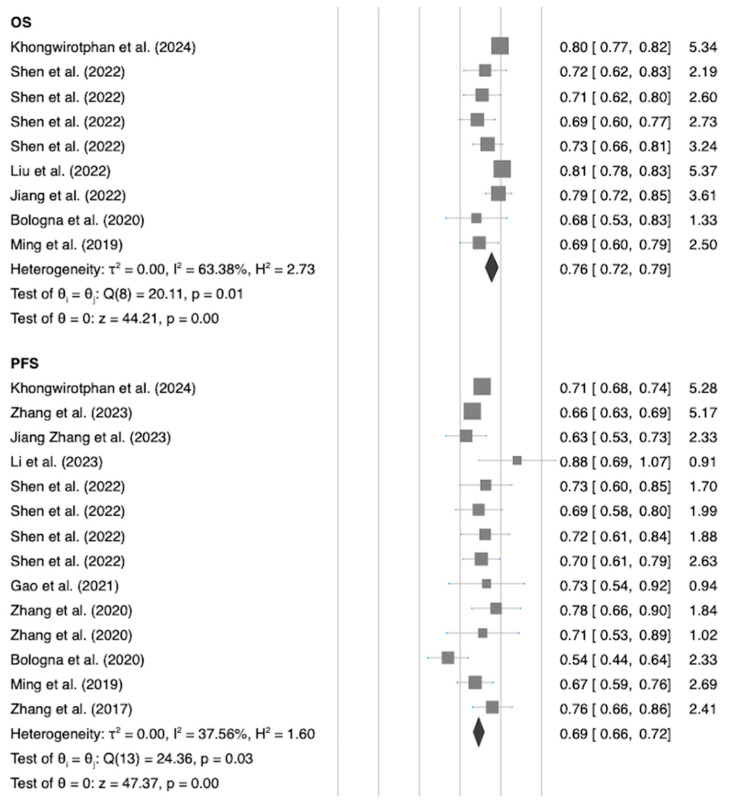
Forest plot of subgroup analysis of radiomics prognosis models’ c-indexes with endpoint as moderator [21,22,23,24,25,26,27,28,29,30,31,32,33,34,35].

In our evaluation of 37 radiomics prognostic outcomes, we observed a concordance index (c-index) ranging from 54% to 81%. The average c-index was a robust 72% (95% Confidence Interval (CI): 70–74%), as depicted in Figure 3. The Q-test yielded a 129.43 (*p* < 0.01), indicating significant heterogeneity. The Higgins I^2^ statistic confirmed moderate heterogeneity, accounting for 64.44% variance, further supported by our sensitivity analysis (Figure S1). The Egger test showed no significant publication bias (*p* = 0.14), as illustrated in the funnel plot (Figure S2). Subgroup analysis revealed significant differences (*p* = 0.03) across endpoints.

For Distant Metastasis-Free Survival (DMFS), we noted a c-index of 0.72 (95% CI: 0.68–0.75), with low variability (I^2^ = 38.76%). Local Recurrence-Free Survival (LRFS) demonstrated a c-index of 0.70 (95% CI: 0.66–0.74, *p* = 0.89) and showed no heterogeneity (I^2^ = 0%). Progression-Free Survival (PFS) had a c-index of 0.69 (95% CI: 0.66–0.72), with low heterogeneity (I^2^ = 37.56%). Lastly, Overall Survival (OS) presented a c-index of 0.76 (95% CI: 0.72–0.79), with moderate heterogeneity (I^2^ = 63.38%). These findings highlight the variability in the consistency of radiomics prognostic models across different oncological endpoints.

### 3.5. Secondary Outcome: Overall Survival Prediction of Radiomics Prognosis Model

Given that Overall Survival (OS) was the only endpoint associated with moderate heterogeneity, we conducted further subgroup analyses and meta-regression to identify potential moderators that could explain this heterogeneity. Significant differences were observed when considering the validation method and radiomics software as moderators (see Table 4). Specifically, subgroups based on radiomics software exhibited low heterogeneity, although caution is advised due to potential bias from the small number of studies included [131]. This observation warrants confirmation with additional studies.

**Figure 4:**
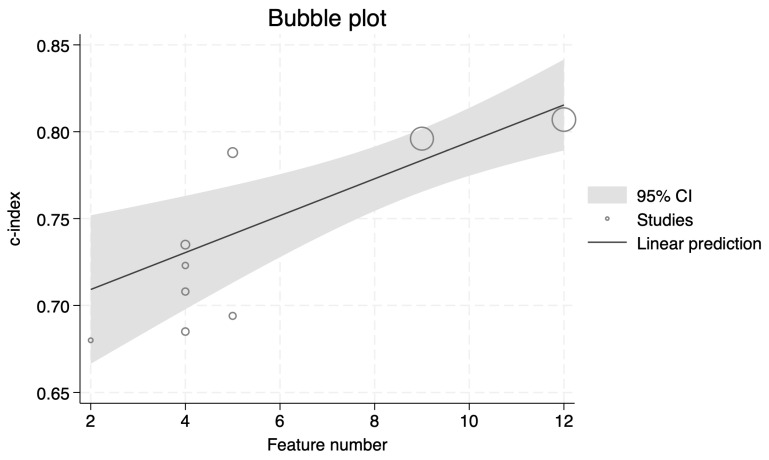
Bubble plot of feature number on radiomics prognosis models with Overall Survival as endpoint.

In our meta-regression analysis, a significant association (*p* < 0.01) was found between the number of features in the prognosis model and its performance, with a coefficient of 0.010622 (Figure 4). No significant association was found with the publication year (coefficient = 0.0220509, *p* = 0.084). There was also no significant relationship between training size (coefficient = 2.72 × 10^−6^, *p* = 0.985) and model performance. 

## 4. Discussion

### 4.1. Overview of Key Findings

Our meta-analysis systematically evaluated 37 radiomics prognostic outcomes, revealing a notable average concordance index (c-index) of 72% (95% Confidence Interval (CI): 70–74%) across studies, with the range stretching from 54% to 81%. This variation not only underscores the potential utility of radiomics in clinical prognostication but also highlights the substantial heterogeneity encountered, particularly in Overall Survival (OS) predictions, where a moderate Higgins I^2^ statistic of 64.44% was observed. Notably, our findings identified a significant positive correlation between the number of features in the prognosis model and its performance, with a meta-regression coefficient of 0.010622 (*p* < 0.01), emphasizing the complexity and potential of detailed models. The analysis also demonstrated that validation methods and radiomics software significantly influenced heterogeneity, pinpointing crucial areas for standardization and improvement in future research.

### 4.2. Comparison with Existing Literature

When compared with the existing literature, our findings both validate the recognized potential of radiomics and highlight the challenges of achieving consistent performance across studies. Notably, the c-index range we report aligns with those found in similar meta-analyses, such as a c-index of 0.762 (95% CI, 0.687–0.837) for Progression-Free Survival (PFS) prediction [13]. Similarly, we limited our analysis to prognosis models incorporating radiomics features. However, we further refined our approach by categorizing endpoints into more sophisticated subgroups, leading to observed reductions in heterogeneity for Local Recurrence-Free Survival (LRFS), Distant Metastasis-Free Survival (DMFS), and PFS. Additionally, our study aggregated results for Overall Survival (OS), an analysis not conducted in the prior study. The moderate heterogeneity observed in OS predictions (I^2^ = 64.44%) suggests that OS may be influenced by a broader range of clinical conditions, potentially necessitating the inclusion of additional clinical features for more robust prediction models.

Our analysis also advances the discussion on methodological variables—specifically, the number of features in a model and the selection of radiomics software. These factors have been less frequently quantified in prior reviews. By highlighting these aspects, our study underscores the need for a more standardized approach to radiomics model development, potentially leading to more consistent and reliable prognostic tools.

### 4.3. Implications for Clinical Practice and Research

The significance of our findings is emphasized through the substantial average concordance index, indicating that radiomic models harbor the potential to considerably refine patient stratification and the planning of treatments. Nonetheless, the observed diversity and fluctuations in performance, especially concerning Overall Survival (OS) predictions, mandate a prudent integration into clinical guidelines. 

The observed variability in the consistency of radiomics prognostic models across different oncological endpoints has important implications for clinical practice. The finding that models performed more consistently for Local Recurrence-Free Survival (LRFS), Distant Metastasis-Free Survival (DMFS), and Progression-Free Survival (PFS) compared to OS suggests that radiomics may be particularly useful for predicting locoregional control and disease progression. This could help clinicians identify high-risk patients who may need more aggressive local therapies or closer surveillance. However, the greater variability in performance for OS indicates that radiomics alone may not be as reliable for predicting long-term survival, which is impacted by many factors beyond the primary tumor.

These results underscore the need to carefully consider the specific clinical endpoint of interest when developing and applying radiomics prognostic models. Models that perform well for one endpoint may not necessarily generalize to other endpoints. Clinicians should look for models that have been validated for the specific outcomes most relevant to their patients and practice. The variability across endpoints also highlights the importance of incorporating other clinical, pathologic, and genomic factors alongside radiomics to develop more holistic prognostic models, particularly for OS. Radiomics can provide valuable information about the primary tumor, but integrating it with other key determinants of survival may be necessary to maximize prognostic value.

Crucially, our detailed subgroup analysis and meta-regression reveals distinct moderators (for instance, validation methodologies profoundly affecting heterogeneity and radiomic software that reduces subgroup heterogeneity) that, upon standardization, could streamline the enhancement and validation of radiomic models. This standardization is pivotal for augmenting their reliability and applicability within clinical frameworks, thus facilitating improved patient care and treatment outcomes. 

Despite these promising avenues, moderate heterogeneity in OS predictions persists, highlighting the complex interplay between radiomic data and patient-specific clinical factors such as health status, comorbidities, and response to treatment. The multifactorial nature of OS suggests that while radiomics can provide valuable insights into tumor characteristics, a comprehensive approach that integrates radiomic data with clinical parameters is essential for making more accurate prognostic assessments. Moreover, variations in treatment protocols and intrinsic tumor heterogeneity contribute further to the observed disparities in survival predictions.

Finally, the results suggest that further research is needed to understand the biological underpinnings of the radiomics features that drive prognostic performance for different endpoints. Better mechanistic insight could help refine models and identify radiomics signatures that are more specifically linked to the most clinically meaningful outcomes. Ongoing research to refine and integrate radiomics into multifaceted prognostic models will be key to realizing its potential to guide precision oncology care.

### 4.4. Technical Considerations of Radiomics Features and Imaging Protocols

Standardized approaches have been implemented to address the potential risk of bias due to protocol variations in radiomics studies to ensure consistency and comparability across different cancer pathologies. Khanfari et al. [132] employed a standardized dataset alongside consistent preprocessing techniques, including normalization and enhancement across various mpMRI images. This method was vital for minimizing data handling variability and included the use of uniform fusion techniques and robust preprocessing methods, which are essential in prostate cancer grading and reducing bias from data processing variations. Similarly, Reginelli et al. [133] standardized the radiomics pipeline by using consistent image acquisition protocols and radiomics software, thus enhancing the reliability of their findings and mitigating the risk of bias across studies. 

To further ensure the relevance and accuracy of prognosis models, statistical techniques and machine learning were used for selecting radiomics features. Methods like the Least Absolute Shrinkage and Selection Operator (LASSO), recursive feature elimination, and correlation analyses (Pearson and Spearman) identified features with minimal redundancy. The reproducibility and consistency of these features were evaluated using intraclass and interclass correlation coefficients. Univariate and multivariate analyses, including Cox regression, further refined the selection based on statistical significance and clinical relevance.

Additionally, robust radiomics software was utilized to normalize data across different MRI scanner settings, mitigating the impact of scanning variability on feature extraction and model performance. MRI sequences were categorized into single and multiple sequences to examine how sequence variations affect the predictive power of radiomics features. This structured approach clarified the influence of technical variations in MRI on radiomic analysis and enhanced the results’ reliability and applicability. These comprehensive measures effectively addressed potential biases due to protocol variations, leading to more reliable and applicable outcomes in radiomics studies.

### 4.5. Methodological Considerations and Strengths

The foundational strength of our study lies in its methodological precision, highlighted by a meticulous systematic review and exhaustive analyses, including the use of meta-regression to identify sources of heterogeneity. The employment of established evaluation tools such as the Quality in Prognosis Studies (QUIPS) and the Radiomics Quality Score (RQS) enhances the reliability of our findings. QUIPS provides a qualitative assessment of bias across various domains of prognostic studies, adding depth to our analysis, while the RQS offers a quantitative measure of methodological quality. Higher RQS scores denote studies with lower risks of bias and greater methodological reliability, essential for ensuring the validity and reproducibility of results. This scoring system not only aids in distinguishing high-quality studies but also pinpoints areas needing improvement in study design and execution.

Moreover, the integration of RQS in a meta-regression against study results allows for a nuanced exploration of how methodological quality impacts reported outcomes in radiomics research. Our findings from the meta-regression, showing a coefficient of −0.0083655 with a *p*-value of 0.294, indicate no significant association between RQS scores and study outcomes at conventional levels of statistical significance. This analysis underscores the importance of robust methodological design in influencing the findings of radiomics studies and provides a reproducible framework for future research in this evolving field.

### 4.6. Limitations and Future Research Directions

Notwithstanding the compelling nature of our results, they are accompanied by limitations. The marked heterogeneity (I^2^ = 64.44% for OS), potential biases, and paucity of studies within certain subgroups reflect the intricate nature of radiomics research and might temper the strength of our deductions. The inability to include unpublished studies raises the possibility of publication bias, while heterogeneity in patient populations, treatments, endpoints, and radiomics methods may limit the reliability and generalizability of pooled estimates. The retrospective nature of the included studies, lack of prospective validation, and absence of a direct assessment of the clinical utility of radiomics compared to standard prognostic tools are also important limitations that underscore the need for ongoing research.

Future inquiries should focus on conducting multi-institutional prospective studies to validate radiomics models in diverse patient cohorts and real-world settings. Methodological standardization, integration of radiomics with other prognostic factors, and mechanistic investigations are key priorities. Rigorous assessments of the clinical utility and impact of integrating radiomics into prognostic models and treatment strategies are essential. Expanding radiomics research to other cancer types and imaging modalities, as well as fostering multidisciplinary collaboration and data sharing, will be crucial for advancing the field. Such endeavors are pivotal for bridging the gap between radiomics research and its clinical application, ultimately leading to more effective and personalized treatment approaches. By addressing these challenges and opportunities, future research can help transform radiomics from a promising research tool into a validated and impactful asset for advancing precision oncology.

## 5. Conclusions

In summary, our meta-analysis highlighted the significance and variability of radiomics in predicting cancer treatment outcomes, particularly focusing on overall survival due to its heterogeneity. 

## Figures and Tables

**Table 1:** Basic characteristics of studies.

Author	Geographic	Validation Method	Study Design	Study Duration	Patients (Train/Valid)	Age	Sex (Male)	Stage	Treatment	Endpoint
Khongwirotphan et al. (2024) [21]	Thailand	Internal validation	Retrospective	2010~2019	183 (146/37)	50 (42.5–57.5)—median/IQR	143 (78.14%)	I~IVA	IMRT, IMRT + AC	DMFS, PFS, OS
Qihao Zhang et al. (2023) [22]	China	Internal validation	Retrospective	2018~2020	151 (75/76)	Train: 52.0 ± 11.2; Test: 49.1 ± 11.4—mean/std	Train: 56 (74.7%); Test: 58 (76.3%)	I~IV	IMRT	PFS
Jiang Zhang et al. (2023) [23]	China	External validation	Retrospective	2013~2019	469 (286/183)	Train: 53; Test: 55—median	Train: 216 (75.5%); Test: 143 (78.1%)	II~IV	CCRT	DFS
Li et al. (2023) [24]	China	Internal validation	Retrospective	2018~2021	145 (102/43)	Train: 49 (42–57); Test: 51 (40–57)—median	Train: 75 (73.5%); Test: 29 (67.4%)	I~IV	IMRT	PFS
Hu et al. (2023) [25]	China	External validation	Retrospective	2008~2017	1072 (575/497)	Train: 44 (38–51); Test: 48 (40–56)—median	Train: 395 (68.7); Test: 326 (65.6)	II	IMRT	DMFS
Shen et al. (2022) [26]	China	External validation	Retrospective	2013~2016	893 (390/503)	Train: 48.0 (40.0–56.3); I: 48.0 (40.5–57.5); E1: 47.0 (42.0–53.0); E2: 49.0 (40.0–57.0); E3: 48.0 (42.0–54.0)—median	Train: 276 (70.8); I: 98 (76.0); E1: 76 (73.8); E2: 101 (71.6); E3: 94 (72.3)	III~IVA	IC + CCRT or CCRT + AC	PFS, LRFS, DMFS, OS
Liu et al. (2022) [27]	China	Internal validation	Retrospective	2013~2021	504 (353/151)	Train: 49.22(9–85); Test: 47.59(10–80)—mean/range	Train: 243 (68.8); Test: 106 (70.2)	I~IVA	IC, CCRT	OS
Li et al. (2022) [28]	China	Internal validation	Retrospective	2010~2012	778 (518/260)	Train: 44 (38–53); Test: 46 (38–52)—median/IQR	Train: 371 (71.6%); Test: 194 (74.6%)	I~IVA	RT alone, CCRT, IC + CCRT	DMFS
Jiang et al. (2022) [29]	China	Internal validation	Retrospective	2015~2017	218 (173/45)	46.1 ± 11.4—mean/std	164 (75.2%)	I~IV	IC, CCRT, AC	OS
Gao et al. (2021) [30]	China	Internal validation	Retrospective	2012~2014	316 (237/79)	NR	Train: 164 (69.2%); Test: 56 (70.9%)	III~IV	Concurrent radical chemoradiotherapy	PFS
Zhang et al. (2020) [31]	China	External validation	Retrospective	2014~2017	220 (132/88)	Train: 48 (19–83); I: 49 (27–78); E: 44 (24–70)—median (range)	Train: 96 (72.73%); I: 33 (75.00%); E: 31 (70.45%)	I~IV	Definitive-intent radiotherapy	FFS
Bologna et al. (2020) [32]	Italy	Internal validation	Retrospective	2004~2017	136 (122/14)	48 (39–57)—median (IQR)	95 (70%)	I~IV	RT alone, Concomitant CHT-RT, Induction CHT + concomitant CHT-RT	OS, PFS, LRFS
Zhang et al. (2019) [33]	China	External validation	Retrospective	2009~2015	737 (360/377)	NR	Train: 270 (75.0); I: 90 (75.0); E: 193 (75.1)	I~IV	IMRT, CCRT with or without IC/AC	LRFS
Ming et al. (2019) [34]	China	Internal validation	Retrospective	2010~2012	303 (200/103)	48.8 ± 12.7—mean (std)	226 (75%)	I~IV	NR	DFS, OS, DMFS
Zhang et al. (2017) [35]	China	Internal validation	Retrospective	2007~2013	118 (88/30)	43 ± 10.98—mean (std)	92 (0.78)	III~IV	NR	PFS

Abbreviation: NR: not recorded; IQR: inter quantile range; std: standard deviation; IMRT: intensity modulated radiation therapy; IC: induction chemotherapy; AC: adjuvant chemotherapy; CCRT: concurrent chemoradiotherapy; RT: radiation therapy; CHT: chemotherapy; LRFS: Local Recurrence-Free Survival; DMFS: Distant Metastasis-Free Survival; PFS: Progression-Free Survival; DFS: Disease-Free Survival; FFS: Failure-Free Survival; OS: Overall Survival.

**Table 2:** MRI scanning details.

Author	Slice Thickness	Tesla	Sequence	Scanner
Khongwirotphan et al. (2024) [21]	4 mm	1.5 T	T1w, T2w	GE Signa HDxt
Qihao Zhang et al. (2023) [22]	4 mm	3 T	T1w, dynamic contrast-enhanced, Proton density	Simens Skyra
Jiang Zhang et al. (2023) [23]	3~4 mm	Train: 1.5 T/3 T; Test: 3 T	T1c	Train: Siemens Avanto; Test: GE SIGNA EXCITE, Siemens Avanto, Siemens Skyra Philips Achieva
Li et al. (2023) [24]	4 mm	3 T	proton density-weighted, dynamic contrast-enhanced, T1c	Siemens Skyr
Hu et al. (2023) [25]	4~6 mm	Train: 1.5 T/3 T; Test: 1.5 T/3 T	T1w, T2w, T1c	Train: GE Signa CV/I, Siemens Magnetom Tim Trio; Test: GE Signa Excite 1.5T HD Twin Speed, Philips Achieva 3.0T, Philips Intera 3.0T
Shen et al. (2022) [26]	NR	NR	T1w, T1c	NR
Liu et al. (2022) [27]	4~5 mm	3 T	T1w, T2w, T1c	Simens MAGNETOM Verio
Li et al. (2022) [28]	5 mm	1.5 T/3 T	T1w, T2w, T1c	GE Signa CV/I; Siemens Magnetom Tim Trio
Jiang et al. (2022) [29]	6 mm	1.5 T	T1w, T2w, T1c	GE Signa EXCITE
Gao et al. (2021) [30]	3 mm	1.5 T	T1c	SIEMENS
Zhang et al. (2020) [31]	3~5 mm	1.5 T/3 T	T1w, T2wFS, T1cFS	Train: MAGNETOM Verio; Test: MAGNETOM Verio, Simens Avanto
Bologna et al. (2020) [32]	NR	1.5 T	T1w, T2w	
Zhang et al. (2019) [33]	5~6 mm	1.5 T	T1w, T2w, T1c	Train: GE Signa EXCITE, GE Signa HDx, SIEMENS Espree; Test: SIEMENS Novus15
Ming et al. (2019) [34]	6 mm	1.5 T	T1c	GE Signa
Zhang et al. (2017) [35]	4 mm	1.5 T	T2, T1c	GE Signa EXCITE HD

Abbreviation: NR: Not recorded; FS: Fat suppression.

**Table 3:** Summary of details of and prognosis model.

Author	Segmentation Software	Annotator	Radiomics Software	Feature Selection	Feature Count	Radiomics Feature	Model	Result
Khongwirotphan et al. (2024) [21]	Eclipse software https://journals.plos.org/plosone/article?id=10.1371/journal.pone.0298111 accessed on 23 April 2024	Radiation oncologists	Pyradiomics v4.11	interobserver variability test, univariate analysis with recursive feature elimination	OS: 9; PFS: 1; DMFS: 10	OS: T2_original_shape_MinorAxisLength; T2_wavelet_HLH_glszm_LargeAreaEmphasis; T1_wavelet_LHL_firstorder_Energy; T1_original_shape_Sphericity; T1_wavelet_LLH_glcm_MaxiumProbability; T2_wavelt_LHL_ngtdm_Coarseness; T2_wavelet_LHL_firstorder_Median; T2_wavelet_LHL_gldtm_DependenceNonUniformity; T2_wavelet_HLL_gldm_DependenceNonUniformity; PFS: T2_wavelet_LHL_ngtdm_coarseness; DMFS: T1_original_shape_sphericity; T2_original_shape_Maxium3DDiameter; T2_wavelet_LHH_LargeAreaEmpasis; T2_wavelet_LHH_glszm_ZoneVariance; T1_original_shape_Maxium3DDiameter; T2_wavelet_LHL_ngtdm_Coarness; T2_wavelet_HHL_ngtdm_Coarness; T1_wavelet_LHL_gldm_DependenceNonUniformity; T1_original_glszm_ZoneEntropy; T1_wavelet_HLH_glszm_LargeAreaGrayLevelEmphasis	Cox proportional hazard	OS: 0.796; PFS: 0.708; DMFS: 0.766
Qihao Zhang et al. (2023) [22]	ITK-SNAP v3.4.0	Radiologist	Pyradiomics https://onlinelibrary.wiley.com/doi/10.1111/cas.15704 accessed on 23 April 2024	LASSO	19	NonUniformity_PrecontrastDCE; SmallAreaEmphasis_PrecontrastDCE; Correlation_PostcontrastDCE (10 s); Minimum_PostcontrastDCE (15 s); 90Percentile_PostcontrastDCE (20 s); DifferenceVariance_PostcontrastDCE (20 s); LowGrayLevelZoneEmphasis_PostcontrastDCE (20 s); Autocorrelation_PostcontrastDCE (25 s); InverseVariance_PostcontrastDCE (25 s); HighGrayLevelEmphasis_PostcontrastDCE (25 s); SmallDependenceEmphasis_PostcontrastDCE (25 s); ShortRunHighGrayLevelEmphasis_PostcontrastDCE (25 s); LowGrayLevelZoneEmphasis_PostcontrastDCE (215 s); ClusterShade_PostcontrastDCE (220 s); ClusterShade_PostcontrastDCE (220 s); 90Percentile_T1Map; Median_T1Map; 90Percentile_ProtonDensityMap; Uniformity_ProtonDensityMap	Cox proportional hazard	PFS: 0.66
Jiang Zhang et al. (2023) [23]	Eclipse Aria v13	Treatment planning system	Pyradiomics v2.2.0	volume dependency, ICC values, feature redundancy, outcome relevancy test	10	log.sigma.5.0.mm.3D_glszm_GrayLevelNonUniformity; wavelet.HLH_glszm_ZoneEntropy; wavelet.HLL_glszm_ZoneEntropy; wavelet.LLH_glszm_ZoneEntropy; original_shape_Sphericity; log.sigma.4.0.mm.3D_gldm_DependenceEntropy; wavelet.HHH_glrlm_LowGrayLevelRunEmphasis; log.sigma.5.0.mm.3D_gldm_LowGrayLevelEmphasis; original_firstorder_Kurtosis; log.sigma.2.0.mm.3D_glrlm_RunEntropy	Cox proportional hazard	DFS: 0.63
Li et al. (2023) [24]	ITK-SNAP v3.6.0	Radiologist	Pyradiomics v3.0.1	Pearson correlation analysis, LASSO, backward feature elimination	8	PDw_wavelet-LHL_glszm_LAHGLE; PDw_lbp_firstorder_Variance from; T1c_wavelet-HHH_glszm_LAHGLE; T1c_squareroot_firstorder_RMAD; Ktrans_maximal correlation coefficient; Ktrans_gray level non-uniformity normalized; Kep_complexity; Kep_large dependence high gray level emphasis	Cox proportional hazard	PFS: 0.808
Hu et al. (2023) [25]	3D-Slicer v4.9.0	Oncologist, Radiologist	Pyradiomics v2.1.2	Pearson correlation, LASSO	23	NR	Cox proportional hazard	DMFS: 0.71
Shen et al. (2022) [26]	in-house software https://www.sciencedirect.com/science/article/pii/S0167814022002225 accessed on 23 April 2024	Radiologist	in-house software https://www.sciencedirect.com/science/article/pii/S0167814022002225 accessed on 23 April 2024	intraclass correlation coefficients, univariate analysis, Pearson correlation coefficient, Boruta	4	NR	Cox proportional hazard	PFS: 0.726/0.691/0.723/0.704; LRRFS: 0.691/0.698/0.678/0.684; DMFS: 0.721/0.686/0.736/0.696; OS: 0.723/0.708/0.685/0.735
Liu et al. (2022) [27]	AccuContour software v3.0	Radiologist	Pyradiomics https://www.ncbi.nlm.nih.gov/pmc/articles/PMC9021720/ accessed on 23 April 2024	LASSO	12	T1.original.shape.Maximum2DDiameterRow; T1.wavelet.LLH.glcm.SumAverage; T1.wavelet.LHL.glcm.JointAverage; T1C.original.shape.MeshVolume; T1C.wavelet.LHL.firstorder.Median, T1C.wavelet.HLL.glcm.InverseVariance; T1C.wavelet.HLH.glszm.LargeAreaLowGrayLevelEmphasis; T2.wavelet.LLH.ngtdm.Coarseness; T2.wavelet.LHL.glcm.InverseVariance; T2.wavelet.LHL.glcm.MaximumProbability; T2.wavelet.LHH.firstorder.Maximum, T2.wavelet.HHL.glcm.MaximumProbability	Cox proportional hazard	OS: 0.807
Li et al. (2022) [28]	AnalyzePro https://www.ncbi.nlm.nih.gov/pmc/articles/PMC8983880/ accessed on 23 April 2024	Radiologist	Pyradiomics https://www.ncbi.nlm.nih.gov/pmc/articles/PMC8983880/ accessed on 23 April 2024	interclass correlation coefficient, Pearson correlation coefficient, LASSO	21	T1_shape_Sphericity; T1_WLHH_GLSZM_LGLZE; T1_WHHL_GLCM_IMC2; T1_WHHH_GLCM_IMC2; T1_WHLH_GLCM_IMC2; T1_log.sigma.3.0.mm.3D_NGTDM_Strength; T1_WHHH_NGTDM_Contrast; T2_WHLL_GLDM_LDHGLE; T2_log.sigma.5.0.mm.3D_FOS_Skewness; T2_WHHL_GLSZM_SALGLE; T2_logarithm_NGTDM_Coarseness; T2_WLLH_GLCM_IDMN; T1C_WHLL_GLCM_Correlation; T1C_WLLH_GLSZM_SAHGLE, T1C_Gradient_GLCM_IMC1, T1C_Square_GLCM_Correlation; T1C_Gradient_GLSZM_ZE; T1C_square_GLRLM_RE; T1C_log.sigma.3.0.mm.3D_GLSZM_ZE; T1C_WLHL_GLSZM_ZE; T1C_gradient_GLSZM_GLN	Cox proportional hazard	DMFS: 0.711
Jiang et al. (2022) [29]	RadCloud Radiomics Cloud Platform https://bmcmedimaging.biomedcentral.com/articles/10.1186/s12880-022-00902-6 accessed on 23 April 2024	NR	Pyradiomics v2.2.0	interclass correlation coefficient, LASSO	5	T2WI_wavelet.LLL_Glrlm_ShortRunHighGrayLevelEmphasis; T1WI_original_Gldm_DependenceVariance; CE-T1WI_original_Ngtdm_Busyness; CE-T1WI_squareroot_Firstorder_Variance; CE-T1WI_wavelet.LLH_Gldm_LargeDependenceLowGrayLevelEmphasis	Cox proportional hazard	OS: 0.788
Gao et al. (2021) [30]	Matlab v2018b	Radiologist	Pyradiomics https://onlinelibrary.wiley.com/doi/10.1002/hed.26867 accessed on 23 April 2024	LASSO	24	Original-GLCM-Inverse Variance; Original-GLRLM-LRLGLE; Wavelet-HLL-Firstorder-Skewness; Wavelet-HLL-GLCM-Idmn; Wavelet-HLL-GLCM_Imc1; Wavelet-LHL-GLRLM-LRLGLE; Wavelet-LHH-Firstorder-Total Energy; Wavelet-LHH-GLCM-Joint Energy; Wavelet-LHH-GLCM-Idn; Wavelet-LHH-GLCM-Imc1; Wavelet-LLH-Firstorder-Skewness; Wavelet-LLH-GLCM-Imc2; Wavelet-HLH-Firstorder-Kurtosis; Wavelet-HHH-Firstorder-Median; Wavelet-HHH-Firstorder-Total Energy; Wavelet-HHH-Firstorder-Kurtosis; Wavelet-HHH-GLCM-Difference Variance; Wavelet-HHH-GLCM-Idn; Wavelet-HHH-GLCM-Cluster Prominence; Wavelet-HHH-GLCM-LRLGLE; Wavelet-HHL-GLCM-Inverse Variance; Wavelet-HHL-GLCM-Imc1; Wavelet-LLL-GLCM-MCC; Wavelet-LLL-GLCM-Imc2	Cox proportional hazard	PFS: 0.73
Zhang et al. (2020) [31]	ITK-SNAP software v2.2.0	Radiologist	Pyradiomics https://www.ncbi.nlm.nih.gov/pmc/articles/PMC7739087/ accessed on 23 April 2024	intra/inter-class correlation coefficient, univariate analysis, minimal redundancy maximum relevance, and random forest	12	T1w_original_shape_LeastAxisLength; T1w_wavelet.HL_firstorder_Skewness; T2w_log.sigma.2.0.mm.3D_glcm_ClusterShade; T2w_wavelet.HL_glszm_SizeZoneNonUniformityNormalized; T2w_wavelet.HH_glcm_ClusterShade; T2w_original_glszm_SmallAreaLowGrayLevelEmphasis; T2w_log.sigma.3.0.mm.3D_glrlm_LowGrayLevelRunEmphasis; T1c_wavelet.HH_glrlm_LongRunLowGrayLevelEmphasis; T1c_wavelet.HH_glrlm_ShortRunLowGrayLevelEmphasis; T1c_log.sigma.5.0.mm.3D_glcm_ClusterShade; T1c_wavelet.LH_glcm_Autocorrelation; T1c_wavelet.HH_firstorder_Skewness	Cox proportional hazard	FFS: 0.711
Bologna et al. (2020) [32]	NR	Radiologist	Pyradiomics v2.2.0	ICC, Spearman correlation coefficient, univariate and multivariate Cox regression	2	T-T1w-WaveletLLH-Firstorder-Median; T-T1w-WaveletLLL-Firstorder-Mean	Cox proportional hazard	OS: 0.68; DFS: 0.54; LRFS: 0.72
Zhang et al. (2019) [33]	RadiAnt software https://www.ncbi.nlm.nih.gov/pmc/articles/PMC6491646/ accessed on 23 April 2024	Radiologist	Matlab v2016a	recursive feature elimination, univariate and multivariate analysis	11	T2-w_GLCM_Cluster_S_Offset_8_Direction_135; T1-w_GLRLM_SRHGE_Direction_90; T1-w_IntensityDirect_Local_Entropy_Mean; T1-w_GLCM_Inverse_Variance_Offset_2_Direction_45; T1-w_GLCM_Contrast_Offset_4_Direction_90; T1-w_GLCM_Dissimilarity_Offset_4_Direction_90; subtraction_GLCM_Inverse_Variance_Offset_4_Direction_135; subtraction_GLCM_IMC_Offset_2_Direction_90; subtraction_IntensityHistogram_Quantile30; subtraction_NeighborIntensityDifference_Busyness; subtraction_GLCM_Cluster_P_Offset_8_Direction_90	Cox proportional hazard	LRFS: 0.753
Ming et al. (2019) [34]	MIM v6.6	Oncologist	Matlab v2015a	Contour reproducibility and nonredundancy, LASSO	DFS: 5; OS: 3; DMFS: 4	DFS:LL_GLCM.Information_Measures_II; HL_GLCM.Information_Measures_II; LL_HIST.mean; LL_HIST.kurtosis; HH_HIST.median; OS: HL_GLCM.Information_Measures_II; LL_HIST.skewness; LL_HIST.kurtosis; DMFS: LL_GLCM.Information_Measures_II; HL_GLCM.Information_Measures_II; LL_HIST.skewness; LL_HIST.kurtosis	Cox proportional hazard	DFS:0.674; OS: 0.694; DMFS: 0.669
Zhang et al. (2017) [35]	ITK-SNAP https://aacrjournals.org/clincancerres/article/23/15/4259/257519/Radiomics-Features-of-Multiparametric-MRI-as-Novel accessed on 23 April 2024	Radiologist	Matlab v2014a	LASSO	7	CET1-w_5_fos_mean; CET1-w_5_GLCM_correlation; CET1-w_5_GLRLM_RP; T2-w_Max3D; T2-w_3_fos_mean; T2-w_6_GLCM_IMC1; T2-w_1_GLRLM_SRLGLE	Cox proportional hazard	PFS: 0.758

**Table 4:** Subgroup analysis of radiomics prognosis model with Overall Survival as endpoint.

Moderator	N of Studies	c-Index (95% CI)	*p*-Values	% I^2^
Region			0.33	
Asian	8	0.76 (0.72~0.79)	<0.01	70%
Europe	1	0.68 (0.53~0.83)	<0.01	0%
Validation method			0.03	
External	3	0.71 (0.67~0.76)	<0.01	0%
Internal	6	0.78 (0.74~0.81)	<0.01	56%
MRI sequence			0.20	
Multiple	8	0.76 (0.72~0.80)	<0.01	67%
Single	1	0.69 (0.60~0.79)	<0.01	0%
Radiomics software			<0.01	
In-house	5	0.71 (0.67~0.75)	<0.01	1%
Pyradiomics	4	0.80 (0.72~0.79)	<0.01	3%

## Data Availability

Data are contained within the article and Supplementary Files.

## Supplementary Materials

The following supporting information can be downloaded at https://www.mdpi.com/article/10.3390/diagnostics14090924/s1. Figure S1: Sensitivity analysis of radiomics prognosis models’ c-index with leave one out method. Figure S2: Funnel plot of radiomics prognosis models’ c-index group by different endpoint. Table S1. PRISMA Checklist. Table S2. Keywords and search results in different databases. Table S3. Excluded article and reason. Table S4. Details of QUIPS assessment. Table S5. Details of radiomic quality score.
