# Supplementary material for: Deciphering the Prognostic Efficacy of MRI Radiomics in Nasopharyngeal Carcinoma: A Comprehensive Meta-Analysis
Source: Diagnostics (Basel). 2024 Apr 29;14(9):924. doi: 10.3390/diagnostics14090924 (PMC11082984; doi:10.3390/diagnostics14090924)
Supplement: Supplementary file 1 [file diagnostics-14-00924-s001.zip › diagnostics-2910129-supplementary.pdf]

Deciphering the Prognostic Efficacy of MRI Radiomics in Nasopharyngeal Carcinoma: A Comprehensive Meta-Analysis

Chih-Keng Wang, Ting-Wei Wang, Chia-Fung Lu, Yu-Te Wu, Man-Wei Hua

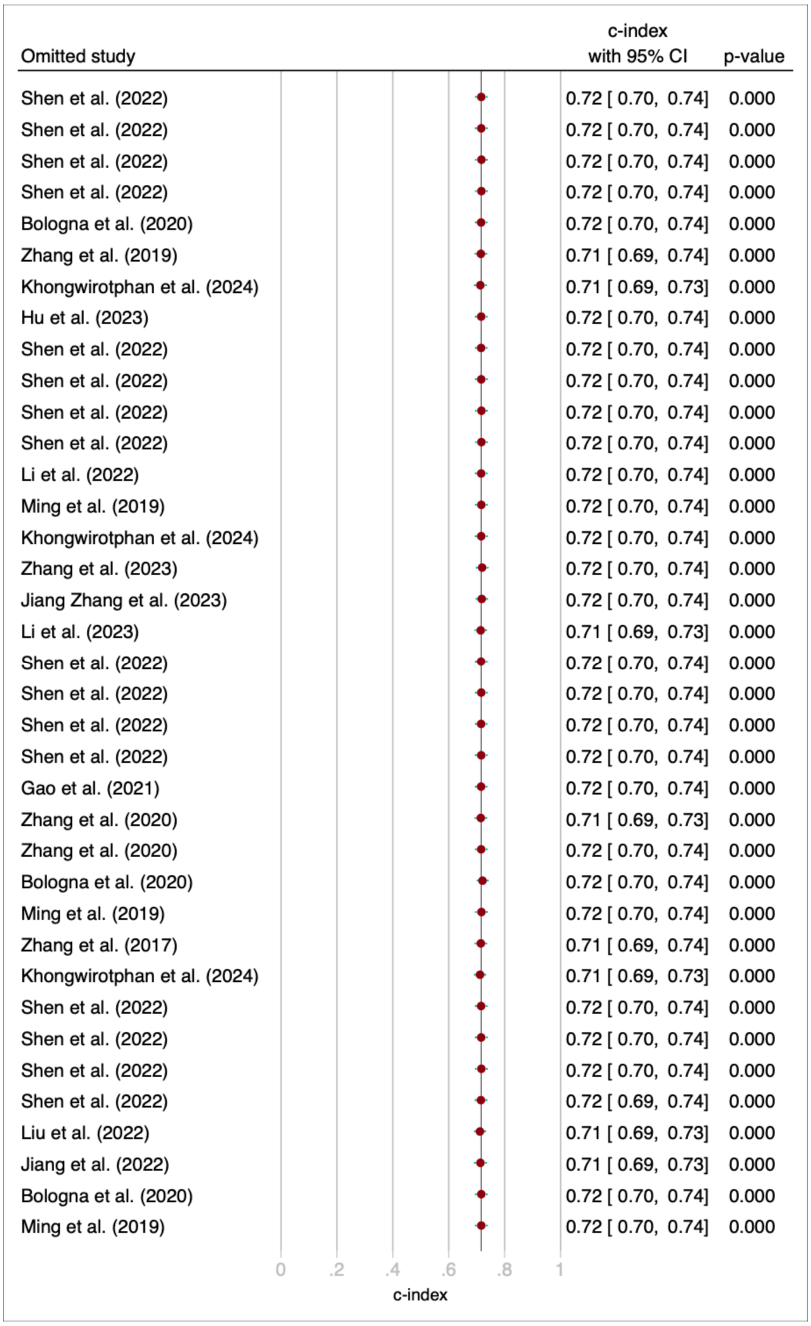

Figure S1: Sensitivity analysis of radiomics prognosis models' c-index with leave one out method.

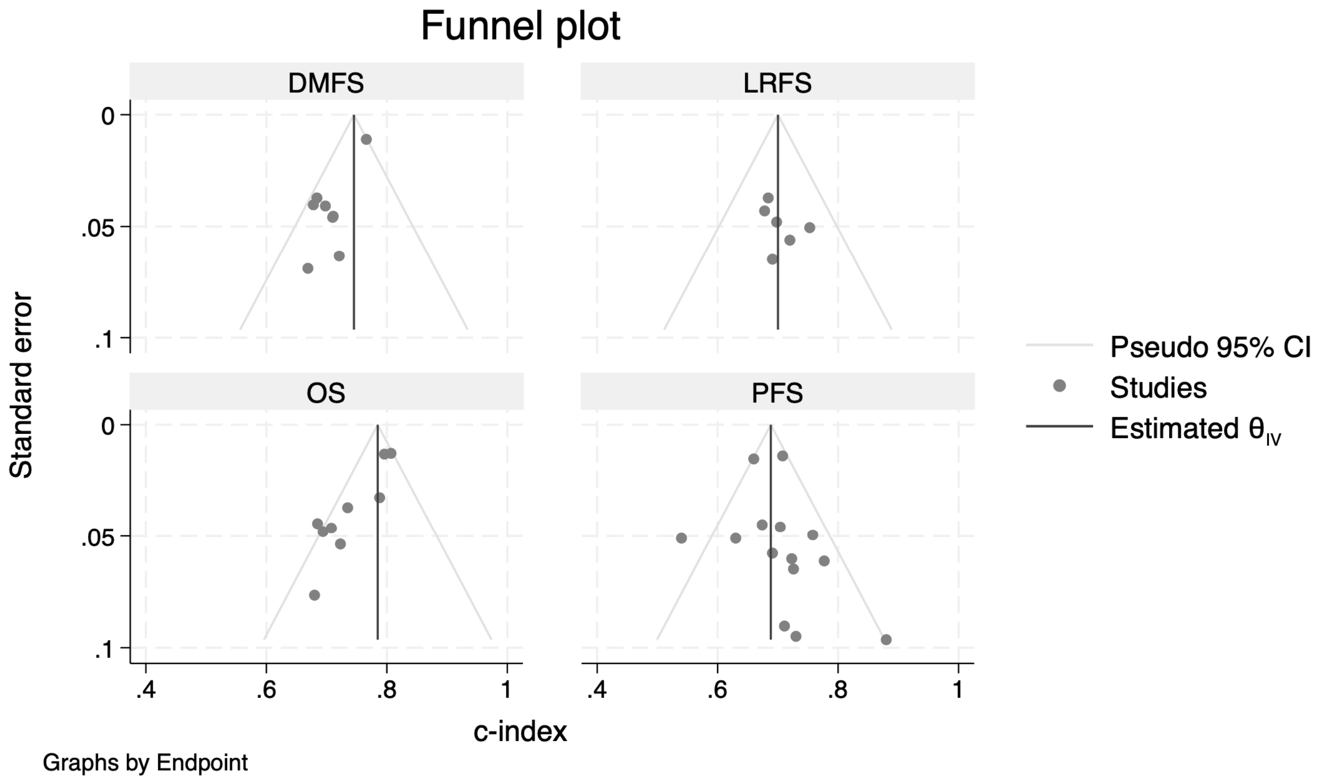

Figure S2: Funnel plot of radiomics prognosis models' c-index group by different endpoint (Egger test: p=0.14)

Table S1. PRISMA Checklist

**Section and Topic**	**Item#**	**Checklist item**	Location where item is reported
		TITLE	
Title	1	Identify the report as a systematic review.	Title
		ABSTRACT	
Abstract	2	See the PRISMA 2020 for Abstracts checklist.	Abstract
		INTRODUCTION	
Rationale	3	Describe the rationale for the review in the context of existing knowledge.	Introduction
Objectives	4	Provide an explicit statement of the objective(s) or question(s) the review addresses.	Introduction
		METHODS	
Eligibility criteria	5	Specify the inclusion and exclusion criteria for the review and how studies were grouped for the syntheses.	Methods
Information sources	6	Specify all databases, registers, websites, organizations, reference lists and other sources searched or consulted to identify studies. Specify the date when each source was last searched or consulted.	Methods
Search strategy	7	Present the full search strategies for all databases, registers, and websites, including any filters and limits used.	Methods
Selection process	8	Specify the methods used to decide whether a study met the inclusion criteria of the review, including how many reviewers screened each record and each report retrieved, whether they worked independently, and if applicable, details of automation tools used in the process.	Methods
Data collection process	9	Specify the methods used to collect data from reports, including how many reviewers collected data from each report, whether they worked independently, any processes for obtaining or confirming data from study investigators, and if applicable, details of automation tools used in the process.	Methods
Data items	10a	List and define all outcomes for which data were sought. Specify whether all results that were compatible with each outcome domain in each study were sought (e.g. for all measures, time points, analyses), and if not, the methods used to decide which results to collect.	Methods,
	10b	List and define all other variables for which data were sought (e.g. participant and intervention characteristics, funding sources). Describe any assumptions made about any missing or unclear information.	Methods
Study risk of bias assess-ment	11	Specify the methods used to assess risk of bias in the included studies, including details of the tool(s) used, how many reviewers assessed each study and whether they worked independently, and if applicable, details of automation tools used in the process.	Methods
Effect measures	12	Specify for each outcome the effect measure(s) (e.g. risk ratio, mean difference) used in the synthesis or presentation of results.	Methods
Synthesis methods	13a	Describe the processes used to decide which studies were eligible for each synthesis (e.g. tabulating the study intervention characteristics and comparing against the planned groups for each synthesis (item #5)).	Methods
	13b	Describe any methods required to prepare the data for presentation or synthesis, such as handling of missing summary statistics, or data con-versions.	Methods
	13c	Describe any methods used to tabulate or visually display results of individual studies and syntheses.	Methods
	13d	Describe any methods used to synthesize results and provide a rationale for the choice(s). If meta-analysis was performed, describe the model(s), method(s) to identify the presence and extent of statistical heterogeneity, and software package(s) used.	Methods
	13e	Describe any methods used to explore possible causes of heterogeneity among study results (e.g. subgroup analysis, meta-regression).	Methods
	13f	Describe any sensitivity analyses conducted to assess robustness of the synthesized results.	Methods
Reporting bias assessment	14	Describe any methods used to assess risk of bias due to missing results in a synthesis (arising from reporting biases).	Methods
Certainty assessment	15	Describe any methods used to assess certainty (or confidence) in the body of evidence for an outcome.	Not applicable

Section and Topic	Item#	Checklist item	Location where item is reported
		RESULTS	
Study selection	16a	Describe the results of the search and selection process, from the number of records identified in the search to the number of studies included in the review, ideally using a flow diagram.	Results, Figure 1
	16b	Cite studies that might appear to meet the inclusion criteria, but which were excluded, and explain why they were excluded.	Table S3
Study characteristics	17	Cite each included study and present its characteristics.	Results, Table 1-3
Risk of bias in studies	18	Present assessments of risk of bias for each included study.	Figure 2, Table S4-5
Results of individual studies	19	For all outcomes, present, for each study: (a) summary statistics for each group (where appropriate) and (b) an effect estimate and its precision (e.g. confidence/credible interval), ideally using structured tables or plots.	Results, Figure 3
Results of syntheses	20a	For each synthesis, briefly summarise the characteristics and risk of bias among contributing studies.	Results, Table S4-5
	20b	Present results of all statistical syntheses conducted. If meta-analysis was done, present for each the summary estimate and its precision (e.g. confidence/credible interval) and measures of statistical heterogeneity. If comparing groups, describe the direction of the effect.	Results, Figure 3-4, Table 4
	20c	Present results of all investigations of possible causes of heterogeneity among study results.	Results, Figure 3-4, Table 4
	20d	Present results of all sensitivity analyses conducted to assess the robustness of the synthesized results.	Figure S1
Reporting biases	21	Present assessments of risk of bias due to missing results (arising from reporting biases) for each synthesis assessed.	Figure S2
Certainty of evidence	22	Present assessments of certainty (or confidence) in the body of evidence for each outcome assessed.	Not applicable
		DISCUSSION	
Discussion	23a	Provide a general interpretation of the results in the context of other evidence.	Discussion
	23b	Discuss any limitations of the evidence included in the review.	Discussion
	23c	Discuss any limitations of the review processes used.	Discussion
	23d	Discuss implications of the results for practice, policy, and future research.	Discussion
		OTHER INFORMATION	
Registration and protocol	24a	Provide registration information for the review, including register name and registration number, or state that the review was not registered.	Methods
	24b	Indicate where the review protocol can be accessed, or state that a protocol was not prepared.	Methods
	24c	Describe and explain any amendments to information provided at registration or in the protocol.	Not applicable
Support	25	Describe sources of financial or non-financial support for the review, and the role of the funders or sponsors in the review.	Funding
Competing interests	26	Declare any competing interests of review authors.	Conflict of interest
Availability of data, code and other materials	27	Report which of the following are publicly available and where they can be found: template data collection forms; data extracted from in-cluded studies; data used for all analyses; analytic code; any other materials used in the review.	Result

Table S2. Keywords and search results in different databases

Database	Keyword	Date	Results
PubMed	((Nasopharyngeal Neoplasms OR Nasopharyngeal Cancer OR Nasopharyngeal Carcinoma OR Nasopharyngeal Tumors) AND (MRI OR magnetic resonance imaging OR MR) AND (machine learning OR deep learning OR radiomics OR texture analysis) AND (overall survival OR disease-free survival OR progression-free survival OR local recurrence OR local control OR local failure OR survival OR prognosis OR prediction OR treatment outcome OR prognostic))	2024/2/17	122
Embase	((Nasopharyngeal Neoplasms OR Nasopharyngeal Cancer OR Nasopharyngeal Carcinoma OR Nasopharyngeal Tumors) AND (MRI OR magnetic resonance imaging OR MR) AND (machine learning OR deep learning OR radiomics OR texture analysis) AND (overall survival OR disease-free survival OR progression-free survival OR local recurrence OR local control OR local failure OR survival OR prognosis OR prediction OR treatment outcome OR prognostic))	2024/2/17	237
Web of Science	((Nasopharyngeal Neoplasms OR Nasopharyngeal Cancer OR Nasopharyngeal Carcinoma OR Nasopharyngeal Tumors) AND (MRI OR magnetic resonance imaging OR MR) AND (machine learning OR deep learning OR radiomics OR texture analysis) AND (overall survival OR disease-free survival OR progression-free survival OR local recurrence OR local control OR local failure OR survival OR prognosis OR prediction OR treatment outcome OR prognostic))	2024/2/17	136

Table S3 Excluded article and reason.

Article	Exclusion reason
Early prediction of long-term survival of patients with nasopharyngeal carcinoma by multi-parameter MRI radiomics	Insufficient detail of c-index
Multi-omics fusion with soft labeling for enhanced prediction of distant metastasis in nasopharyngeal carcinoma patients after radiotherapy	Outcome insufficient for quantitative meta-analysis
Significance of radiologic extranodal extension in locoregionally advanced nasopharyngeal carcinoma with lymph node metastasis: a comprehensive nomogram	Not radiomics
Deep Learning-Based Multi-Modality Segmentation of Primary Gross Tumor Volume in CT and MRI for Nasopharyngeal Carcinoma	Outcome does not relate to interest
Integrative Scoring System for Survival Prediction in Patients With Locally Advanced Nasopharyngeal Carcinoma: A Retrospective Multicenter Study	Insufficient detail of c-index
Proposed prognostic subgroups and facilitated clinical decision-making for additional locoregional radiotherapy in de novo metastatic nasopharyngeal carcinoma: a retrospective study based on recursive partitioning analysis	Outcome insufficient for quantitative meta-analysis
A deep learning MRI-based signature may provide risk-stratification strategies for nasopharyngeal carcinoma	Deep learning based radiomics

Intra- and peritumoral MRI radiomics assisted in predicting radiochemotherapy response in metastatic cervical lymph nodes of nasopharyngeal cancer	Insufficient detail of c-index
Multimodality radiomics analysis based on [18F]FDG PET/CT imaging and multisequence MRI: application to nasopharyngeal carcinoma prognosis	Not MRI
Adjuvant chemotherapy or no adjuvant chemotherapy? A prediction model for the risk stratification of recurrence or metastasis of nasopharyngeal carcinoma combining MRI radiomics with clinical factors	Insufficient detail of c-index
MRI-based clinical radiomics nomogram may predict the early response after concurrent chemoradiotherapy in locally advanced nasopharyngeal carcinoma	Insufficient detail of c-index
Delta-Radiomics Guides Adaptive De-Intensification after Induction Chemotherapy in Locoregionally Advanced Nasopharyngeal Carcinoma in the IMRT Era	Delta radiomics
Radiomic analysis of MRI for prediction of response to induction chemotherapy in nasopharyngeal carcinoma patients	Insufficient detail of c-index
Explainable machine learning via intra-tumoral radiomics feature mapping for patient stratification in adjuvant chemotherapy for locoregionally advanced nasopharyngeal carcinoma	Outcome does not relate to interest
A nomogram model based on pre-treatment and post-treatment MR imaging radiomics signatures: application to predict progression-free survival for nasopharyngeal carcinoma	Outcome insufficient for quantitative meta-analysis
Deep learning-based precise prediction and early detection of radiation-induced temporal lobe injury for nasopharyngeal carcinoma	Outcome does not relate to interest
Artificial intelligence aided precise detection of local recurrence on MRI for nasopharyngeal carcinoma: a multicenter cohort study	Insufficient detail of c-index
Deep learning-based accurate delineation of primary gross tumor volume of nasopharyngeal carcinoma on heterogeneous magnetic resonance imaging: A large-scale and multi-center study	Outcome does not relate to interest
Prognosis Forecast of Re-Irradiation for Recurrent Nasopharyngeal Carcinoma Based on Deep Learning Multi-Modal Information Fusion	Insufficient detail of c-index
Predictive function of tumor burden-incorporated machine-learning algorithms for overall survival and their value in guiding management decisions in patients with locally advanced nasopharyngeal carcinoma	Deep learning based radiomics
Radiomic signatures reveal multiscale intratumor heterogeneity associated with tissue tolerance and survival in re-irradiated nasopharyngeal carcinoma: a multicenter study	Insufficient detail of c-index
Prognostic model on overall survival in elderly nasopharyngeal carcinoma patients: a recursive partitioning analysis identifying pre-treatment risk stratification	Outcome insufficient for quantitative meta-analysis
A Rulefit-based prognostic analysis using structured MRI report to select potential beneficiaries from induction chemotherapy in advanced nasopharyngeal carcinoma: A dual-centre study	Not radiomics
Automatic tumor segmentation and metachronous single-organ metastasis prediction of nasopharyngeal carcinoma patients based on multi-sequence magnetic resonance imaging	Insufficient detail of c-index
Performance of Pretreatment MRI-Based Radiomics in Recombinant Human Endostatin Plus Concurrent Chemoradiotherapy Response Prediction in Nasopharyngeal Carcinoma: A Retrospective Study	Insufficient detail of c-index

Deep learning for the prediction of residual tumor after radiotherapy and treatment decision-making in patients with nasopharyngeal carcinoma based on magnetic resonance imaging	Insufficient detail of c-index
Deep Learning for Predicting Distant Metastasis in Patients with Nasopharyngeal Carcinoma Based on Pre-Radiotherapy Magnetic Resonance Imaging	Outcome insufficient for quantitative meta-analysis
Intravoxel incoherent motion radiomics nomogram for predicting tumor treatment responses in nasopharyngeal carcinoma	Outcome does not relate to interest
Deep learning-based recurrence detector on magnetic resonance scans in nasopharyngeal carcinoma: A multicenter study	Insufficient detail of c-index
MRI-based radiomics models can improve prognosis prediction for nasopharyngeal carcinoma with neoadjuvant chemotherapy	Outcome insufficient for quantitative meta-analysis
Radiomics for Predicting Response of Neoadjuvant Chemotherapy in Nasopharyngeal Carcinoma: A Systematic Review and Meta-Analysis	Review article
Establishment and validation of novel MRI radiomic feature-based prognostic models to predict progression-free survival in locally advanced rectal cancer	Not nasopharyngeal cancer
Prediction of Response to Induction Chemotherapy Plus Concurrent Chemoradiotherapy for Nasopharyngeal Carcinoma Based on MRI Radiomics and Delta Radiomics: A Two-Center Retrospective Study	Outcome insufficient for quantitative meta-analysis
Extraction parameter optimized radiomics for neoadjuvant chemotherapy response prognosis in advanced nasopharyngeal carcinoma	Insufficient detail of c-index
MRI-based random survival Forest model improves prediction of progression-free survival to induction chemotherapy plus concurrent Chemoradiotherapy in Locoregionally Advanced nasopharyngeal carcinoma	Outcome insufficient for quantitative meta-analysis
A Clinical-Radiomics Nomogram Based on Magnetic Resonance Imaging for Predicting Progression-Free Survival After Induction Chemotherapy in Nasopharyngeal Carcinoma	Outcome insufficient for quantitative meta-analysis
MRI-Based Back Propagation Neural Network Model as a Powerful Tool for Predicting the Response to Induction Chemotherapy in Locoregionally Advanced Nasopharyngeal Carcinoma	Insufficient detail of c-index
Dynamic contrast-enhanced magnetic resonance imaging-based radiomics for the prediction of progression-free survival in advanced nasopharyngeal carcinoma	Overlapping dataset
Deep learning for locally advanced nasopharyngeal carcinoma prognostication based on pre- and post-treatment MRI	Deep learning based radiomics
Multi-Organ Omics-Based Prediction for Adaptive Radiation Therapy Eligibility in Nasopharyngeal Carcinoma Patients Undergoing Concurrent Chemoradiotherapy	Insufficient detail of c-index
Radiomics based on pretreatment MRI for predicting distant metastasis of nasopharyngeal carcinoma: A preliminary study	Insufficient detail of c-index
Deep learning-enabled precise recurrence detection in nasopharyngeal carcinoma: A multicentre study	Deep learning based radiomics
Machine Learning Based on MRI DWI Radiomics Features for Prognostic Prediction in Nasopharyngeal Carcinoma	Insufficient detail of c-index
MRI-based radiomics nomogram for predicting temporal lobe injury after radiotherapy in nasopharyngeal carcinoma	Outcome does not relate to interest
A diagnosis model in nasopharyngeal carcinoma based on PET/MRI radiomics and semiquantitative parameters	Not MRI

Integration of MRI-Based Radiomics Features, Clinicopathological Characteristics, and Blood Parameters: A Nomogram Model for Predicting Clinical Outcome in Nasopharyngeal Carcinoma	Insufficient detail of c-index
Add-on individualizing prediction of nasopharyngeal carcinoma using deep-learning based on MRI: A multicentre, validation study	Deep learning based radiomics
A MRI-based radiomics model predicting radiation-induced temporal lobe injury in nasopharyngeal carcinoma	Outcome does not relate to interest
Baseline MRI-based radiomics model assisted predicting disease progression in nasopharyngeal carcinoma patients with complete response after treatment	Insufficient detail of c-index
A deep learning-based radiomic nomogram for prognosis and treatment decision in advanced nasopharyngeal carcinoma: A multicentre study	Deep learning based radiomics
MRI-Based Deep-Learning Model for Distant Metastasis-Free Survival in Locoregionally Advanced Nasopharyngeal Carcinoma	Insufficient detail of c-index
The usefulness of pretreatment mr-based radiomics on early response of neoadjuvant chemotherapy in patients with locally advanced nasopharyngeal carcinoma	Insufficient detail of c-index
MRI-based radiomics as response predictor to radiochemotherapy for metastatic cervical lymph node in nasopharyngeal carcinoma	Insufficient detail of c-index
A Prognostic Predictive System Based on Deep Learning for Locoregionally Advanced Nasopharyngeal Carcinoma	Deep learning based radiomics
Unambiguous advanced radiologic extranodal extension determined by MRI predicts worse outcomes in nasopharyngeal carcinoma: Potential improvement for future editions of N category systems	Outcome insufficient for quantitative meta-analysis
Whole-Tumor Histogram and Texture Imaging Features on Magnetic Resonance Imaging Combined With Epstein-Barr Virus Status to Predict Disease Progression in Patients With Nasopharyngeal Carcinoma	Insufficient detail of c-index
A Gene-Expression Predictor for Efficacy of Induction Chemotherapy in Locoregionally Advanced Nasopharyngeal Carcinoma	Not radiomics
Early risk-assessment of patients with nasopharyngeal carcinoma: the added prognostic value of MR-based radiomics	Insufficient detail of c-index
Predictive Value of a Combined Model Based on Pre-Treatment and Mid-Treatment MRI-Radiomics for Disease Progression or Death in Locally Advanced Nasopharyngeal Carcinoma	Insufficient detail of c-index
Application Value of Magnetic Resonance Radiomics and Clinical Nomograms in Evaluating the Sensitivity of Neoadjuvant Chemotherapy for Nasopharyngeal Carcinoma	Insufficient detail of c-index
Magnetic resonance imaging-based radiogenomics analysis for predicting prognosis and gene expression profile in advanced nasopharyngeal carcinoma (vol 43, pg 3730, 2021)	Overlapping dataset
An interpretable machine learning prognostic system for locoregionally advanced nasopharyngeal carcinoma based on tumor burden features	Not radiomics
Prognostic and predictive value of radiomics features at MRI in nasopharyngeal carcinoma	Outcome insufficient for quantitative meta-analysis
A deep learning MR-based radiomic nomogram may predict survival for nasopharyngeal carcinoma patients with stage T3N1M0	Deep learning based radiomics
Deep learning-based prognosis prediction in T3N1 nasopharyngeal carcinoma patients treated with induction chemotherapy followed by concurrent chemoradiotherapy	Deep learning based radiomics

MRI-based radiomics nomogram may predict the response to induction chemotherapy and survival in locally advanced nasopharyngeal carcinoma	Outcome insufficient for quantitative meta-analysis
Pretreatment MRI-Derived Radiomics May Evaluate the Response of Different Induction Chemotherapy Regimens in Locally advanced Nasopharyngeal Carcinoma	Insufficient detail of c-index
Multi-sequence MRI based Radiomics Model in Predicting Efficacy of Neoadjuvant Chemotherapy for Nasopharyngeal Carcinoma	Insufficient detail of c-index
A predictive model of radiation-related fibrosis based on the radiomic features of magnetic resonance imaging and computed tomography	Outcome does not relate to interest
Predicting Progression-Free Survival Using MRI-Based Radiomics for Patients With Nonmetastatic Nasopharyngeal Carcinoma	Overlapping dataset
Deep learning for risk prediction in patients with nasopharyngeal carcinoma using multi-parametric MRIs	Outcome insufficient for quantitative meta-analysis
Radiomics Analysis and Correlation With Metabolic Parameters in Nasopharyngeal Carcinoma Based on PET/MR Imaging	Not MRI
Machine Learning Analysis of Image Data Based on Detailed MR Image Reports for Nasopharyngeal Carcinoma Prognosis	Insufficient detail of c-index
Exploring MRI based radiomics analysis of intratumoral spatial heterogeneity in locally advanced nasopharyngeal carcinoma treated with intensity modulated radiotherapy	Outcome does not relate to interest
Radiomics on multi-modalities MR sequences can subtype patients with non-metastatic nasopharyngeal carcinoma (NPC) into distinct survival subgroups (vol 29, pg 1211, 2019)	Overlapping dataset
Radiomic Nomogram: Pretreatment Evaluation of Local Recurrence in Nasopharyngeal Carcinoma based on MR Imaging	Without standalone radiomics result
Development and validation of a magnetic resonance imaging-based model for the prediction of distant metastasis before initial treatment of nasopharyngeal carcinoma: A retrospective cohort study	Insufficient detail of c-index
Pretreatment Prediction of Adaptive Radiation Therapy Eligibility Using MRI-Based Radiomics for Advanced Nasopharyngeal Carcinoma Patients	Insufficient detail of c-index
A multidimensional nomogram combining overall stage, dose volume histogram parameters and radiomics to predict progression-free survival in patients with locoregion-ally advanced nasopharyngeal carcinoma	Without standalone radiomics result
Magnetic Resonance Imaging Texture Analysis Predicts Recurrence in Patients With Nasopharyngeal Carcinoma	Insufficient detail of c-index
Deep learning in nasopharyngeal carcinoma: A retrospective cohort study of 3D convolutional neural networks on magnetic resonance imaging	Outcome does not relate to interest
Predictive value of pretreatment MRI texture analysis in patients with primary nasopharyngeal carcinoma	Without independent validation
Radio-Transcriptomic Phenotypes Predict Radioresistance in Nasopharyngeal Carcinoma	Outcome does not relate to interest
Comparison of radiomics tools for image analyses and clinical prediction in nasopharyngeal carcinoma	Outcome does not relate to interest
Use of radiomics in the recurrence patterns after IMRT for head and neck cancer: a preliminary study	Outcome insufficient for quantitative meta-analysis
Radiomics model to predict early progression of nonmetastatic nasopharyngeal carcinoma after intensity modulation radiation therapy: A multicenter study	Insufficient detail of c-index

Development and validation of a novel MR imaging predictor of response to induction chemotherapy in locoregionally advanced nasopharyngeal cancer: a randomized controlled trial substudy (NCT01245959)	Outcome does not relate to interest
Development and validation of M1 substages for previously untreated metastatic nasopharyngeal carcinoma	Not radiomics
Multiparametric MRI Based Radiomics for the Prediction of Induction Chemotherapy Response and Survival in Locally Advanced Nasopharyngeal Carcinoma	Outcome insufficient for quantitative meta-analysis
Pretreatment MR imaging radiomics signatures for response prediction to induction chemotherapy in patients with nasopharyngeal carcinoma	Insufficient detail of c-index
Predicting chemoradiotherapy response of nasopharyngeal carcinoma using texture features based on intravoxel incoherent motion diffusion-weighted imaging	No combination of features to the overall model
Use of Radiomics Combined With Machine Learning Method in the Recurrence Patterns After Intensity-Modulated Radiotherapy for Nasopharyngeal Carcinoma: A Preliminary Study	Outcome insufficient for quantitative meta-analysis
Advanced nasopharyngeal carcinoma: pre-treatment prediction of progression based on multi-parametric MRI radiomics	Insufficient detail of c-index
Radiomic machine-learning classifiers for prognostic biomarkers of advanced nasopharyngeal carcinoma	Insufficient detail of c-index
Association of MRI radiomics feature changes with treatment outcome for radiotherapy of nasopharyngeal carcinoma	Outcome does not relate to interest
Exploration and validation of radiomics signature as an independent prognostic biomarker in stage III-IVb nasopharyngeal carcinoma	Outcome does not relate to interest
MRI based radiomics signature, a quantitative prognostic biomarker for nasopharyngeal carcinoma	Overlapping dataset
Use of texture analysis based on contrast-enhanced MRI to predict treatment response to chemoradiotherapy in nasopharyngeal carcinoma	Outcome insufficient for quantitative meta-analysis
Classification of Progression Free Survival with Nasopharyngeal Carcinoma Tumors	Outcome insufficient for quantitative meta-analysis

Table S4 Details of QUIPS assessment

Author	Study Participant	Study attribution	Prognosis factor measurement	Outcome measurement	Study confounding	Statistical analyses and reporting
Khongwirotphan et al. (2024) [14]	Low	Low	Low	Low	Unclear	Low
Qihao Zhang et al. (2023) [15]	Low	Low	Low	Low	Unclear	Low
Jiang Zhang et al. (2023) [16]	Low	Low	Low	Low	High	Low
Li et al. (2023) [17]	Low	Low	Low	Low	Unclear	Low
Hu et al. (2023) [18]	Low	Low	Low	Low	High	Low
Shen et al. (2022) [19]	Low	Unclear	Low	Low	Low	Low
Liu et al. (2022) [20]	Low	Low	Low	Low	Low	Low
Li et al. (2022) [21]	Low	Low	Low	Low	Low	Low
Jiang et al. (2022) [22]	Low	Low	Low	Low	Low	Low
Gao et al. (2021) [23]	Low	Low	Low	Low	Low	Low
Zhang et al. (2020) [24]	Low	Low	Low	Low	Low	Low
Bologna et al. (2020) [25]	Low	Unclear	Low	Low	Unclear	Low
Zhang et al. (2019) [26]	Low	Low	Low	Low	High	Low
Ming et al. (2019) [27]	Low	Low	Low	Low	Low	Low
Zhang et al. (2017) [28]	Low	Low	Low	Low	Low	Low

Table S5 Details of radiomic quality score.

Author	Image protocol quality	Multiple segmentation	Phantom study on all scanner	Imaging at multiple time points	Feature reduction or adjustment for multiple testing	Multivariable analysis with non-radiomics features	Detect and discuss biological correlates	Cut-off analyses	Discrimination statistics	Calibration statistics	Prospective study registered in a trial database	Validation	Comparison to 'gold standard'	Potential clinical utility	Cost-effectiveness analysis	Open science and data	Total
Khongwirotphan et al. (2024) [14]	1	1	0	0	3	1	0	1	1	0	0	2	0	0	0	1	11
Qihao Zhang et al. (2023) [15]	1	0	0	0	3	0	1	1	1	0	0	2	0	0	0	0	9
Jiang Zhang et al. (2023) [16]	1	0	0	0	3	0	0	1	1	0	0	3	0	0	0	1	10
Li et al. (2023) [17]	1	0	0	0	3	0	1	1	1	0	0	2	0	0	0	0	9
Hu et al. (2023) [18]	1	0	0	0	3	1	0	1	1	0	0	4	0	0	0	2	13
Shen et al. (2022) [19]	0	1	0	1	3	0	0	1	1	1	0	5	1	1	0	0	15
Liu et al. (2022) [20]	1	0	0	0	3	0	0	1	1	1	0	2	0	1	0	1	11
Li et al. (2022) [21]	1	0	0	0	3	1	0	1	1	1	0	2	0	1	0	0	11
Jiang et al. (2022) [22]	1	1	0	1	3	1	0	1	1	1	0	2	0	1	0	1	14
Gao et al. (2021) [23]	1	1	0	0	3	1	0	1	1	1	0	2	0	0	0	0	11
Zhang et al. (2020) [24]	1	0	0	0	3	1	1	1	1	1	0	3	0	0	0	0	12
Bologna et al. (2020) [25]	1	0	0	0	3	1	0	1	1	0	0	2	0	0	0	0	9
Zhang et al. (2019) [26]	1	1	0	0	3	1	0	1	1	1	0	3	0	0	0	0	12
Ming et al. (2019) [27]	1	1	0	0	3	1	0	1	1	0	0	2	0	0	0	0	10
Zhang et al. (2017) [28]	1	1	0	0	3	1	0	1	1	1	0	2	0	0	0	0	11
